# Trends, Gaps, and Challenges in Antiviral Resistance: A Bibliometric Analysis and Narrative Synthesis of Key Viruses, Resistance Mechanisms, and Drivers

**DOI:** 10.1155/bmri/3771603

**Published:** 2026-10-08

**Authors:** Uwem Okon Edet, Md Zulfekar Ali, Bridget Enuwa Abah, Ebere Roseann Agusi, Olanrewaju Igah, Adeboyejo Kazeem Adeyinka, Clement Meseko

**Affiliations:** ^1^ Infectious and Transboundary Animal Diseases National Veterinary Research Institute (NVRI) Vom Nigeria nvri.gov.ng; ^2^ Microbiology Department School of Pure and Applied Sciences Federal University of Technology Ikot Abasi Akwa Ibom State Nigeria futo.edu.ng; ^3^ Animal Health Research Division Bangladesh Livestock Research Institute Savar Dhaka Bangladesh blri.gov.bd; ^4^ Department of Medical Microbiology and Parasitology Faculty of Basic Clinical Science Olabisi Onabanjo University Ago Iwoye Ogun State Nigeria oouagoiwoye.edu.ng

**Keywords:** antiviral resistance, landscape, mechanisms, trajectory, viral

## Abstract

**Background:**

Ribonucleic acid (RNA) viruses mutate faster than bacteria and most DNA viruses, yet our understanding of mutation‐induced viral resistance remains limited compared with bacterial resistance. This has resulted in a fragmented global research landscape on antiviral resistance.

**Aim:**

This study is aimed at providing a holistic understanding of the global research landscape of antiviral resistance using bibliometric approach and narrative synthesis.

**Methods:**

Both PubMed and Scopus database was systematically searched following the Scientific Procedures and Rationales for Systematic Literature Reviews (SPAR‐4‐SLR) protocol. Scopus yielded 375 records, whereas PubMed yielded 100 documents, all of which completely overlapped with those retrieved from Scopus. Given this overlap, the final dataset from Scopus (*n* = 314) was analyzed using VOSviewer, Biblioshiny, and R to identify publication trends, keyword evolution, author productivity, institutional contributions, and collaborative networks. Additionally, key findings from the Top 20 most cited studies and research on resistance mechanisms were synthesized to highlight research gaps.

**Results:**

A total of 314 documents published between 1991 and 2026 (March 23, 2026) across 194 sources were identified, showing a modest annual growth rate of 2%. Research output peaked in 2020, although citation patterns were inconsistent. Keywords were dominated by pathogens such as human immunodeficiency virus (HIV), influenza, and COVID‐19, indicating a focus on pathogen. Publications were concentrated in a limited number of high‐impact journals (28.03%) and led predominantly by institutions in high‐income countries, especially the United States. Mechanistically, resistance primarily arose from mutations in viral enzymes, with major drivers, including poor adherence, suboptimal dosing, prolonged therapy, misuse of antivirals, and intrinsic viral mutation rates. It is important to note that the mandatory inclusion of One Health–related terms in the search strategy may have excluded relevant antiviral resistance publications that did not explicitly utilize the One Health framework, potentially limiting the comprehensiveness of the retrieved literature.

**Conclusion:**

Antiviral resistance research within the One Health framework remains limited, narrowly focused, and geographically concentrated. Broader, interdisciplinary studies and enhanced global research capacity are urgently needed to address existing knowledge gaps and disparities.

## 1. Introduction

The term “anti‐viral resistance” (AVR) gained increased attention since the 1980s onwards [1, 2], although the precise date of its first use remains unclear. Antiviral resistance as a phenomenon has gained prominence as an important public health issue. Coque et al. [3] identified antimicrobial resistance (AMR) as one of the challenges of the 21st century. Historically, AMR has been largely associated with antibacterial resistance and has consequently received greater attention than viral resistance, primarily because of the widespread use of antibacterial agents and the substantial burden of bacterial AMR [4–8]. This difference in attention is striking, especially given the fact that RNA viruses have a higher mutation rate than most DNA viruses, with both possessing higher abilities to cause pandemics compared with bacteria [9]. Antiviral therapies have improved health outcomes for viral infections, including HIV [10], human cytomegalovirus [11], and herpes simplex virus [12], to mention a few. However, their long‐term efficacy and effectiveness are threatened by the emergence of resistant viral strains, raising concerns about future treatment sustainability. The adverse effects of antiviral resistance are enormous but often receive less attention than antibacterial resistance. These consequences include treatment failure, selection of mutant strains, increased transmission, prolonged hospital stay, and heightened healthcare costs [13–16].

As expected, various studies have emanated in an attempt to understand diverse aspects of antiviral resistance, including genomic analyses of human pathogens such as HIV [17], genetic susceptibility studies of humans to viral infections [18], and genomic surveillance studies [19] to mention a few. As a study design, bibliometric studies can provide temporal quantitative and qualitative insights into a field of study [20]. Specifically, they can reveal trends, research gaps and challenges within a field of study, and have been applied to diverse pathogens, including HIV/acquired immunodeficiency syndrome (AIDS) in Nigeria [21], Lassa virus globally [22], avian influenza globally [23] and in Southeast Asia [24], vaccine uptake in Africa [25], metagenomics in diagnosis and detection of pathogens [26] and monkeypox virus globally [27]. A few bibliometric studies exist that have utilized bibliometric to examine various aspects of antiviral resistance studies. Immanuel et al. [28] evaluated majority and minority hepatitis C virus (HCV) drug‐resistant variants. Putri et al. [29] performed a global bibliometric assessment of antiviral peptide research. On the other hand, Yan et al. [30] examined HIV‐1 drug‐resistant minority variants from 1999 to 2024, whereas Meseko et al. [31] evaluated antiviral options and therapeutics against influenza.

Antiviral resistance is increasingly recognized as a One Health concern because the emergence, selection, persistence, and dissemination of drug‐resistant viruses are driven by interconnected processes occurring across human, animal, and environmental interfaces [32, 33]. Like antibacterial resistance, AVR and resistant viral variants may emerge under antiviral drug pressure in humans and animals and subsequently circulate within human and animal populations, creating challenges for treatment, disease control, and outbreak management [34]. This is particularly relevant for viruses with established animal reservoirs or zoonotic potential, including influenza viruses, coronaviruses, and other emerging viral pathogens, where viral evolution and host switching can drive the emergence and dissemination of variants with altered susceptibility to antiviral agents [35]. The use of antiviral agents in human and veterinary medicine may exert selective pressure that contributes to the emergence or maintenance of resistant variants [34]. Furthermore, interactions among humans and animals (domestic and wildlife) and shared environments create opportunities for viral transmission and genetic evolution that cannot be adequately understood from a human health perspective alone [33]. Surveillance for resistance has taken center stage in clinical research and requires collaboration among clinicians, veterinarians, virologists, epidemiologists, environmental researchers, public health scientists, and policymakers. Therefore, a One Health perspective is important for understanding antiviral resistance as a systems‐level problem involving pathogen evolution, antimicrobial use, host interactions, transmission networks, and environmental interfaces [33].

Despite the existence of studies, these studies have only examined some aspects of the broad subject of antiviral resistance by focusing on HIV variants and antiviral peptides or influenza [28–31]. This leaves the global landscape of antiviral resistance fragmented and poorly understood, especially in terms of its evolution, gaps, trends, challenges and countermeasures. Currently, several important research questions remain regarding antiviral resistance worldwide. These include the following: (1) What are the major trends and their types of AVR research? (2) What core outlets are the favorable destinations of AVR studies? (3) What authors and their affiliations dominate the research landscape? (4) What are the key findings from the Top 20 most cited studies on AVR? (5) How have AVR research themes evolved over time? (6) What pathogens dominate the AVR literature? (7) What is the overall collaboration network on AVR? Given the highlighted gaps, there is an urgent need to examine the research landscape, as such analyses can reveal research priorities and strengthen the global response to AVR. As a significant One Health issue, addressing AVR requires coordinated efforts, including improved antiviral stewardship, enhanced genomic surveillance, development and clinical use of newer and safer antiviral agents and stronger international collaboration networks [3]. If AVR is not addressed, healthcare systems around the globe will remain inadequate and ill‐prepared to handle emerging and re‐emerging viral threats. A global bibliometric analysis of antiviral resistance can help characterize these gaps. Thus, our study is aimed at revealing trends, identifying gaps, analyzing core journals, and examining challenges via analyzing the trajectory of AVR, most productive authors and affiliations, keyword trends and thematic evolution, and synthesis of evidence from the Top 20 most cited studies. Additionally, we will provide a narrative synthesis of the major drivers and risk factors associated with AVR, resistance mechanisms across diverse viral families, and the antiviral agents against which resistance has been reported.

## 2. Methodology

### 2.1. Bibliometric Study Design

As a study design, bibliometric studies are widely used to provide valuable insights into the research landscape of a particular field. Recent studies have applied bibliometric analysis across various fields, including research on zoonotic pathogens of pandemic potential, vaccine uptake and even diagnostic technologies [23–25]. Given that bibliometric studies provide insight into a particular field of study, a successful analysis depends on a comprehensive and clearly defined search strategy. To achieve this, we adopted the Scientific Procedures and Rationales for Systematic Literature Review (SPAR‐4‐SLR) protocol [36] (Figure 1). The adoption of the SPAR‐4‐SLR protocol was intended to guarantee methodological quality and rigor, while ensuring reproducibility.

**Figure 1:**
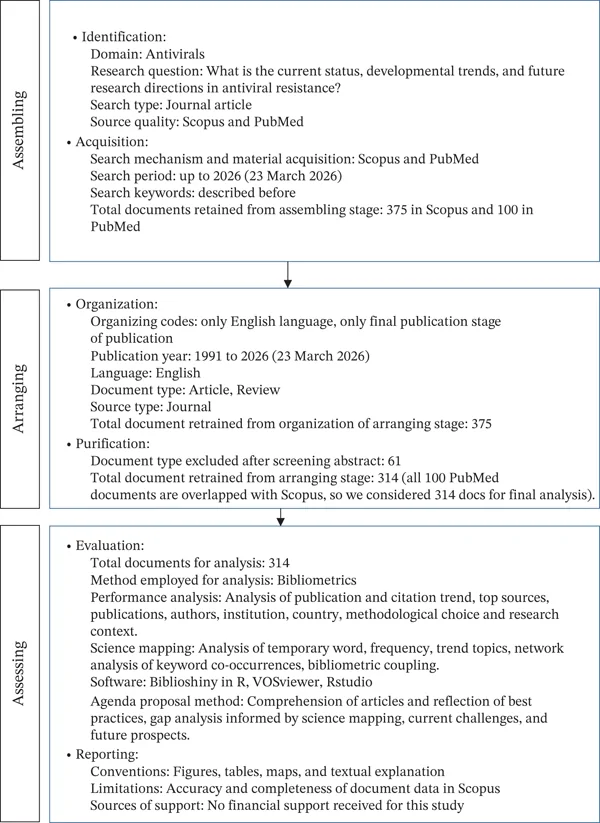
A flowchart description of the SPAR‐4‐SLR protocol showing stages (identification, acquisition, organization, purification, evaluation, and reporting), including the database search strategy, screening process, inclusion criteria, duplicate assessment, and final selection of publications used for the bibliometric analysis.

### 2.2. Database Choice and Screening of Bibliographic Data

For this study, PubMed and Scopus databases were searched. Our rationale for utilizing PubMed as one of our search platform stemmed from the fact that it has provision for other resources, including MEDLINE, a reputable primary database of citations and abstracts and National Library of Medicine resources, making it one of the most widely accessible bibliographic search platforms globally. As a search platform, PubMed hosts medical literature from around the world [37]. On the other hand, we utilized the Scopus database because of its broader multidisciplinary coverage. As a database, Scopus indexes literature in physical sciences, life sciences, health and medical sciences, social sciences, humanities and related disciplines. Furthermore, this broad coverage makes it more suitable for retrieving studies relevant to One Health compared with PubMed [22, 38]. Using both databases, an initial search was conducted using the same search terms to arrival at the most appropriate database for the study in terms of coverage.

### 2.3. Keywords Utilized in the Search in Scopus and PubMed

Both databases were searched on March 23, 2026, by applying the same search keywords to their titles and abstracts fields using almost similar terms. For the Scopus database, the keywords string used were as follows: TITLE‐ABS‐KEY ((“antiviral resistan∗” OR “viral resistan∗” OR “virus resistan∗” OR “antiviral drug resistan∗”) AND (“One Health” OR “One Medicine” OR “Ecohealth” OR “Planetary Health” OR ((human∗ OR animal∗ OR environment∗) W/3 (health OR reservoir∗ OR transmission)))) AND (LIMIT‐TO (DOCTYPE, “ar”) OR LIMIT‐TO (DOCTYPE, “re”)) AND (LIMIT‐TO (PUBSTAGE, “final”)) AND (LIMIT‐TO (LANGUAGE, “English”)). On the other hand, for PubMed, the terms utilized were: ((“antiviral resistan∗” OR “viral resistan∗” OR “virus resistan∗” OR “antiviral drug resistan∗”) AND (“One Health” OR “One Medicine” OR “Ecohealth” OR “Planetary Health” OR ((human∗ OR animal∗ OR environment∗) W/3 (health OR reservoir∗ OR transmission)))).

### 2.4. Search Process, Inclusion Criteria, Screening, and Data Extraction

The search process was guided by the SPAR‐4‐SLR protocol, and was carried out by two authors working independently. Prior to the actual search, trial searches were carried to optimize the extraction of dataset from a One Health perspective. Briefly, as shown in the SPAR‐4‐SLR protocol, the stages utilized include assembling, arranging and assessing. In the assembling stage, the final number of documents was obtained. The conducted searches were done up to March 23, 2026, with no restriction on publication year. The initial search retrieved 375 records from Scopus and 100 records from PubMed. The records from Scopus and PubMed included studies published up to the 23rd of March 2026. Following the assembling stage of the SPAR‐4‐SLR protocol, records from both databases were compared manually using digital object identifiers (DOIs) and article titles to identify overlapping publications. Given that all the retrieved PubMed records (*n* = 100) were also indexed in Scopus, Scopus dataset was adopted as the final dataset for the analysis to avoid duplication and also ensure a uniform dataset.

In the arranging stage, the predefined inclusion criteria were applied. Only studies written in English, published in the final publication stage and not preprints, and indexed as peer‐reviewed journal articles and review articles were included. In addition, conference proceedings, book chapters, editorials, notes, letters, and other nonpeer‐reviewed publication types were excluded. Following application of all the eligibility criteria, 61 records were excluded for not entirely dwelling on AVR but just mentioned it in passing. The assessing stage comprised data extraction and bibliometric evaluation of the retained dataset. Science mapping analyses included keyword frequency, trend topic analysis, temporal keyword evolution, keyword co‐occurrence networks, and bibliographic coupling. A factorial analysis of the Top 50 most occurring terms was conducted using multiple correspondence analysis (MCA). Analyses were performed using the Bibliometrix package (Biblioshiny) in R (Version 4.5.0), RStudio, and VOSviewer (Version 1.6.20). The complete search, screening, selection, and analytical workflow are illustrated in Figure 1. The trend line analysis was fitted with a quartic polynomial model as it captured the nonlinear fluctuations and overall growth trajectory of annual publications across the study period. Annual growth rate was calculated as the compound annual growth rate (CAGR) of scientific publications over the study period, representing the average yearly rate of increase in the number of documents. It was calculated using the formula: Compounded annual growth rate (*%*) = ([Nf/Ni]^[1/t]^ − 1) × 100, where Nf is the number of publications in the final year, Ni is the number of publications in the initial year, and *t* is the number of years between the initial and final publication years.

### 2.5. Narrative Synthesis of Drivers and Risk Factors of AVR and Major Antiviral‐Resistant Viruses and Key Drugs Used in Clinical Practice

The drivers and risk factors of AVR, and the major antiviral‐resistant viruses and key drugs used in clinical practice were derived through a narrative synthesis of the included studies. Recurrent factors associated with antiviral resistance and their mechanisms were extracted, compared across publications, and categorized into major thematic groups based on their underlying mechanisms. In the same vein, viral families, main drug classes, common resistance‐associated mutations (RAMs) or variants and clinical impacts were also done. Data were extracted using a standardized electronic form. Extracted variables included viral family/species, target drug classes, verified RAMs, underlying molecular mechanisms, clinical/epidemiological impacts, and reported drivers or risk factors.

## 3. Results

### 3.1. Search Summary

The summary of the conducted search screening and selection process is presented in Figure 2. The results spanned 1991–2026 (March 23, 2026). A total of 314 documents were published across 194 sources, with a cumulative annual growth rate of 2%. Within this time frame, a total of 2453 authors contributed to the publication volume. Of these, only 25 documents were single‐authored. The average coauthor per document was 8.4. International collaborative coauthorship accounted for 32.8% of the total studies. A total of 792 unique author keywords. The documents contained 62,203 cited references, whereas the average document age and citation per document were 10.8 years and 63.66 citations per document, respectively.

**Figure 2:**
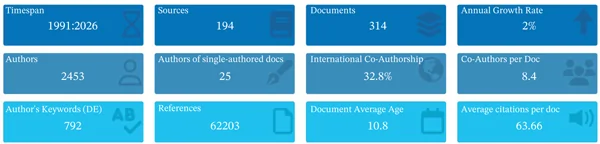
A pictorial summary of the conducted search on AVR in this study.

### 3.2. Study Trajectory on Antiviral Resistance

Figure 3 shows the trajectory of studies covering the period from 1991 to March 23, 2026. The plot reveals a phase of steady output that occurred between 1991 and 2000, with the maximum number of articles being 1. This was followed by a phase of increased publication output that spanned 2001–2014, with number of studies ranging from 2 to 22. Thereafter, the number of studies declined between 2015 and 2018 before increasing to a peak of 27 publications in 2020, the highest annual output on antiviral resistance during the study period. After 2020, publication output fluctuated, ranging from 14 to 23 documents between 2021 and 2024. The fourth‐order (quartic) polynomial regression model equation for the trajectory superimposed on the graph is *y* = 1.48 − 0.932*x* + 0.14*x* ^2^ − 0.00354*x* ^3^ + 1.47 × 10^−5^ *x* ^4^ with a correlation coefficient of 0.66. On the other hand, the number of citations did not reveal any clear pattern. The number of citations for the first decade spanning 1991–2001 ranged from 7 to 94, with the highest recorded in 1994. In the second decade, 2002–2012, the number of citations ranged from 36.5 to 284.3, whereas in the third decade, 2013–2023, the number of citations ranged from 11 to 63.2. The citation counts for 2024 and 2025, ranged from 2.23 to 13.52 per year.

**Figure 3:**
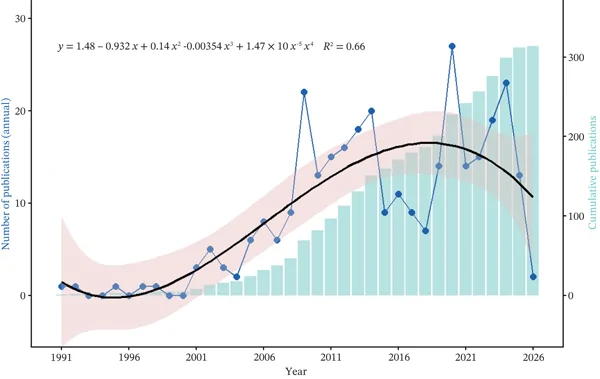
A scatter plot with dual *y*‐axis depicting the number and cumulative publications (blue) plotted in R (Version 4.5.2). As indicated in the plot, each blue dot represents annual number of publications, whereas the shaded area in the plot covering the black trend line indicates the confidence interval. At the top of the graph is a fourth‐order (quartic) polynomial regression model equation and its coefficient of correlation.

### 3.3. Top 20 Most Cited Studies

To provide a broader comprehension of the research landscape, findings from the Top 20 most cited studies addressing AVR were synthesized. The studies covered diverse areas, including viral transmission, epidemiology, pathogenesis, antiviral therapy, and related determinants. The diverse thematic areas from the most cited studies indicate that they contribute to our understanding of AVR. This approach facilitates identification of influential research and interconnected themes that have shaped the AVR research landscape. The top most cited studies are summarized in Table 1 and it shows their DOIs, total citations, total citations per year, and normalized citations. These studies were published between 2002 and 2020. The most cited study was by Thigpen et al. [39] with a citation of 1730. The evidence from these studies is synthesized in the discussion section to show the direction of the most cited studies.

**Table 1:** Top 20 most cited papers on antiviral resistance.

Paper	Total citations (TC)	TC per year	Normalized TC
Antiretroviral Preexposure Prophylaxis for Heterosexual HIV Transmission in Botswana [39]	1730	115.33	6.97
Airborne Transmission of Influenza A/H5N1 Virus Between Ferrets [40]	1446	96.4	5.82
In Vitro and In Vivo Characterization of New Swine‐Origin H1N1 Influenza Viruses [41]	961	53.38	10.88
Avian Influenza Virus (H5N1): A Threat to Human Health. Clinical Microbiology Reviews [42]	846	42.3	2.97
Changing Patterns of Chikungunya Virus: Re‐Emergence of a Zoonotic Arbovirus [43]	650	32.5	2.28
Monkeypox Virus in Nigeria: Infection Biology, Epidemiology, and Evolution [44]	637	91	10.44
Cell‐to‐Cell Spread of HIV Permits Ongoing Replication Despite Antiretroviral Therapy [45]	459	28.68	5.76
Are Ducks Contributing to the Endemicity of Highly Pathogenic H5N1 Influenza Virus in Asia? [46]	414	18.81	4.32
The Interferons and Their Receptors—Distribution and Regulation [47]	397	26.46	1.59
Continuing Challenges in Influenza [48]	343	26.38	8.39
Biological Features of Novel Avian Influenza A (H7N9) Virus [49]	341	24.35	5.39
Two‐Dose Intrapartum/Newborn Nevirapine and Standard Antiretroviral Therapy to Reduce Perinatal HIV Transmission: A Randomized Trial [50]	331	13.24	1.62
Guidelines for Using Antiretroviral Agents Among HIV‐Infected Adults and Adolescents: The Panel on Clinical Practices for Treatment of HIV [51]	312	12.48	1.52
Experimental Infection of Domestic Dogs and Cats With SARS‐CoV‐2: Pathogenesis, Transmission, and Response to Reexposure in Cats [52]	311	44.42	5.10
Small‐Molecule Antiviral *β*‐d‐N 4‐Hydroxycytidine Inhibits a Proofreading‐Intact Coronavirus With a High Genetic Barrier to Resistance [53]	277	34.62	4.82
Avian Influenza Virus Infections in Humans [54]	262	12.47	3.35
Neutralization Escape Variants of Human Immunodeficiency Virus Type 1 Are Transmitted From Mother to Infant [55]	247	11.76	3.16
Acquired Prion Disease: Iatrogenic CJD, Variant CJD, Kuru [56]	206	8.58	2.57
Antiviral Drug Resistance as an Adaptive Process [57]	197	17.90	3.62
Antiviral Resistance and the Control of Pandemic Influenza [58]	183	9.15	0.64

Abbreviation: TC, total citations.

### 3.4. Keyword Analyses and Its Evolution Over Time

Figure 4A is a treemap representation of the Top 50 authors′ keywords used globally in AVR research. The Top 10 most used authors′ keywords in descending order of frequency were HIV, pandemic, antiviral resistance, influenza virus, influenza, resistance, antiretroviral therapy, SARS‐CoV2, avian influenza, and COVID‐19. Specific keywords related to antiviral resistance included antiviral resistance, drug resistance, HIV drug resistance, and drug resistance mutations. Among the Top 20 terms were One Health with a frequency of 5, representing 1.75% of the authors′ keywords. Their occurrence could be as a result of a potential source of search‐term bias. However, its low abundance suggests that its prominence may also reflect its genuine importance within the antiviral research landscape. Among the keywords were terms such antiviral medication, including oseltamivir, baloxavir and antiviral drugs. Figure 4B shows the temporal distribution of the Top 18 keywords in use on AVR from 2006 to 2024. The most used was HIV‐1, and this was followed by pandemic and antiviral resistance. However, the most consistently used keyword with the longest span of usage was avian influenza. Recently used keywords post‐2021 include One Health, influenza A virus, mutation, SARS‐Cov2, and COVID‐19. However, mutation has been used as a keyword for almost a decade.

**Figure 4:**
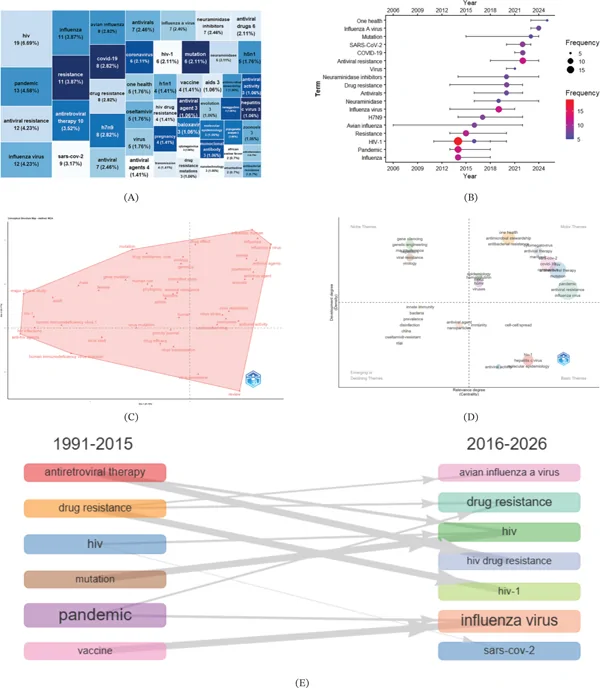
(A–E) Diagrammatic representation of various aspects of the keyword analyses conducted in this study. (A) Tree map of authors′ keywords or keywords plus (Top 50) plotted in RStudio. Each of the tiles represents a keyword, whereas their sizes indicate the frequency of usage, which is indicated underneath each keyword. (B) Temporal trend analysis of the Top 18 keywords showing their frequencies (see legend). The various bubble sizes indicate the frequency of the terms, whereas the straight line across their diameters indicates their temporal distribution. (C) Factorial analysis depicted by a conceptual structure map of the keywords used in AVR research, showing different dimensions constructed in Biblioshiny. The quadrants divide the keywords into four potential thematic areas. (D) Thematic map showing various themes from AVR research. Each quadrant indicates a different theme. Each of the differently colored bubbles indicates subthemes inside a quadrant and their sizes indicate their frequency of occurrence of the keywords (cluster size), reflecting their relative prominence. (E) Thematic evolution of keywords on AVR between two periods: 1991–2015 (left) and 2016–2026 (right). The arrows pointing from left to right show the terms on the left have evolved into terms on the right over time.

Figure 4C shows the various factorial analyses of keywords used in AVR research. The top left quadrant showed the presence of keywords that indicate that studies dealt with antiviral resistance as HIV‐1 dominated. Other keywords in this quadrant related to AVR were virus mutation, gene mutation, mutation, and drug resistance. In the right upper quadrant, terms related to antiviral resistance included resistance and drug effect, whereas virus‐specific and medication‐related terms were influenza and influenza virus. In the bottom left and right quadrants, the number of keywords was fewer and were related to HIV. Figure 4D is a thematic map of terms associated with AVR research. The top quadrant comprised more terms than the bottom quadrant. The top right quadrant comprised motor themes, and the largest cluster included antiviral resistance, pandemic, and influenza virus. In another cluster, the terms were One Health, antimicrobial stewardship and antibacterial resistance. This suggests that their co‐occurrence may partly reflect search‐strategy bias. However, these terms are also intrinsically related to antiviral resistance within the One Health framework and likely reflect the interdisciplinary nature of the field. However, this should be interpreted in light of a methodological limitation, as One Health–related terms were mandatory components of the search strategy. Consequently, the observed association of these terms with AVR may not necessarily reflect a predefined search criterion but rather an emerging interdisciplinary relationship. In the other clusters, diverse terms, including cytomegalovirus and hepatitis C, COVID‐19, antiviral agent and antiviral resistance were present. In the top left cluster, the terms associated with this niche included viral resistance, virology, gene silencing and genetic engineering. Figure 4E shows the thematic evolution of keywords across two periods, 1991–2015 and 2016–2026. Specifically, drug resistance evolved into avian influenza, drug resistance and HIV‐1. Mutation evolved into HIV‐1, pandemic into influenza virus and HIV‐1, antiretroviral therapy evolved into HIV drug resistance and HIV.

### 3.5. Top Publication Outlets and Core Journal Analysis

Table 2 is a summary of the Top 10 journal outlets that published AVR research globally. The results show the number of articles published, their citations, impact factors and quartile rankings. The *Journal of Virology* had the highest number of articles (20), and these were followed by *Clinical Infectious Diseases*, *Journal of Infectious Diseases*, and *PLoS One* with 12 studies each. The citations of the studies were not proportional to the number of articles published as outlets including *Journal of General Virology* and *Viruses* came second and third with citations. All the journals had impact factors that ranged from 2.6 to 7.3, whereas the quartile rankings of the journals ranged from Q1 to Q2. Collectively, the Top 10 outlets published 88 out of the 314 studies, representing 28.03% of the total publications.

**Table 2:** Details of Top 10 journals that published AVR research.

Journals	Articles (%)	Citations	IF (2024)	^∗^Q ranking
Journal of Virology	20 (6.37)	1763	3.8	Q2
Clinical Infectious Diseases	12 (3.82)	724	7.3	Q1
Journal of Infectious Diseases	12 (3.82)	403	4.5	Q2
PLoS One	12 (3.82)	414	2.6	Q2
Antiviral Research	7 (2.23)	315	4.0	Q1
Viruses	7 (2.23)	853	3.5	Q2
Journal of Medical Virology	6 (1.91)	88	4.6	Q1
Antimicrobial Agents and Chemotherapy	4 (1.27)	190	4.5	Q1
Current Topics in Microbiology and Immunology	4 (1.27)	192	4.7	Q2
Journal of General Virology	4 (1.27)	801	4.3	Q1

^∗^Q ranking based on the journal citation reports (JRC) 2024—released in June 2025. Citations represent total citations accumulated by each publication up to the date of literature retrieval.

### 3.6. Institutional Analyses

Table 3 provides a summary of the Top 10 institutions conducting research on AVR globally covering the names, number of documents, total global citations, and total link strength. The Top 7 institutions were the Center for Disease Control and Prevention, National Centre for Immunisation and Respiratory Diseases, University of California, Zhejiang University, St. Jude Children′s Research Hospital, University of North Carolina at Chapel Hill and University of Tokyo with 43, 37, 34, 29, 28, 24, and 23 publications, respectively. The results indicate a few things. First, no African institutions were among the top‐ranked contributors, and representation from low‐ and middle‐income countries was limited, despite the presence of a few institutions from Asia, including Zhejiang University (China). Second, over 70 of the institutions were based in the United States.

**Table 3:** Top 10 leading institutions conducting research on antiviral resistance between 1991 and 2026.

Rank	Institutions	Documents (%)
1	Centers for Disease Control and Prevention, United States	43 (13.69)
2	National Center for Immunization and Respiratory Disease, CDC, United States	37 (11.78)
3	University of California, United States	34 (10.83)
4	Zhejiang University, China	29 (9.24)
5	St. Jude Children′s Research Hospital, United States	28 (8.92)
6	University of North Carolina at Chapel Hill, United States	24 (7.64)
7	University of Tokyo, Japan	23 (7.32)
8	Genus PLC Research and Development, United States	21 (6.69)
9	National Institute of Infectious Diseases	20 (6.37)
10	Harvard School of Public Health, United States	18 (5.73)

Figure 5 is a component bar chart that shows single‐country and multiple‐countries publications (SCP and MCP) authors across the globe conducting AVR research. The United States dominated both SCP and MCP, followed by China and the United Kingdom in second and third places. Egypt featured among SCP and MCP as the only African country. Figure 6 shows the number of citations received by the various countries. The United States dominated the citations, and the same set of countries with SCP and MCP had more citations.

**Figure 5:**
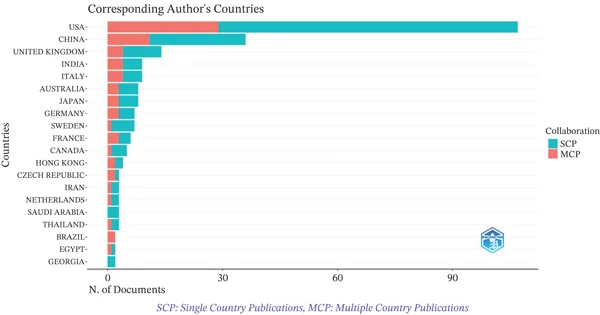
A component bar chart showing countries of correspondence authors (single and multiple publications) across the globe. The blue and red legends indicate the number of single and multiple publications across the various countries.

**Figure 6:**
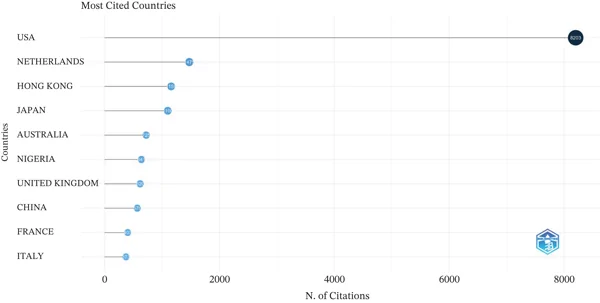
Top 20 most cited countries around the world conducting research on AVR.

### 3.7. Mechanisms, Viral Resistant Families, Drivers, and Risks Factors of Resistance

The mechanisms, representative viral resistant families, drivers and risk factors of resistance were synthesized from included studies and are captured in Table 4. Table 4 summarizes major clinically relevant antiviral‐resistant viruses and their key drugs, focusing on HIV, Influenza virus, SARS‐CoV‐2, and HCV, themes that dominate our bibliometric analysis. The table highlights how RAMs in reverse transcriptase, integrase, neuraminidase, RNA‐dependent RNA polymerase (RdRp), and NS3/4A–NS5A–NS5B proteins compromise the efficacy of NRTIs, NNRTIs, integrase and protease inhibitors, neuraminidase and endonuclease inhibitors, and direct‐acting antivirals (DAAs). These findings indicate that, despite high potency regimens, single or few‐step mutations can still drive treatment failure, particularly in chronic HIV and HCV infections or prolonged exposure (SARS‐CoV‐2 and influenza) settings, underscoring the need for resistance‐guided strategies within broader AMR frameworks.

**Table 4:** Major antiviral‐resistant viruses and key drugs used in clinical practice.

Virus (family)	Main drug class/examples	Common resistance associated mutations or variants	Brief clinical impact	Key references
Human immunodeficiency virus type 1 (HIV‐1) (Retroviridae)	Nucleoside reverse transcriptase inhibitors (NRTIs): zidovudine, lamivudine, tenofovir based regimens; non‐NRTIs: efavirenz, nevirapine; integrase strand transfer inhibitors (INSTIs): dolutegravir, raltegravir; protease inhibitors (PIs): atazanavir, darunavir	NRTI: M184V, K65R; NNRTI: K103N, Y181C; INSTI: R263K, Q148H/R/K; PI: V82A, I84V, G48V, among others	Compromised virological suppression; reduced efficacy of first line; need for regimen switches or resistance guided ART optimization.	[59, 60]
Influenza A virus (Orthomyxoviridae)	Neuraminidase inhibitors (NAIs): oseltamivir, zanamivir; capdependent endonuclease inhibitor: baloxavir marboxil	NA: H275Y (N1 numbering); PA endonuclease: I38T/M/F	Reduced reduction in symptom duration and viral shedding; concern about transmission competent resistant strains in high risk settings.	[59–61]
Severe acute respiratory syndrome coronavirus 2 (SARS‐CoV‐2) (Coronaviridae)	RNA‐dependent RNA polymerase (RdRp) inhibitors: remdesivir; oral RdRp‐targeting agent: molnupiravir; 3CL protease inhibitor: nirmatrelvir (in Paxlovid); monoclonal antibodies (e.g., earlier agents now largely inactive)	RdRp targeting: E802D, C799F, several others under selection; spike protein targeting (mAbs): many S domain mutations (e.g., E484K, N501Y related lineages that evade certain antibodies)	Potential for reduced drug susceptibility and escape from neutralizing antibodies; particular concern in immunocompromised hosts receiving prolonged therapy.	[62, 63]
Hepatitis C virus (Flaviviridae)	Directacting antivirals (DAAs): NS3/4A protease inhibitors (e.g., glecaprevir, voxilaprevir), NS5A inhibitors (e.g., ledipasvir, velpatasvir), NS5B polymerase inhibitors (e.g., sofosbuvir)	NS3/4A: A156T/V, D168A/V; NS5A: Y93H/N, L31M/V; NS5B: S282T (sofosbuvir selected)	Most modern DAA‐based regimens retain high SVR, but selected resistance‐associated substitutions can reduce response in retreatment or complex genotypes; need for genotype tailored salvage regimens.	[64]

In the same vein, the various resistance mechanisms across various families were extracted from the included studies and captured in Table 5. Table 5 outlines major resistance mechanisms across virus families, emphasizing that target‐site mutations in viral enzymes are the predominant pathways in HIV, influenza, SARS‐CoV‐2, and HCV. The result further shows that resistance commonly arises through substitutions in reverse transcriptase, integrase, and protease (HIV), neuraminidase (influenza), RdRp and 3CL‐protease (SARS‐CoV‐2), and NS3/4A, NS5A, and NS5B (HCV), all of which reduce drug binding and enzymatic inhibition. These findings highlight that high‐fidelity inhibitors remain vulnerable to single‐locus mutations, particularly under prolonged or suboptimal drug pressure, reinforcing the need for combination regimens and resistance‐informed design in antiviral development and stewardship.

**Table 5:** Major resistance mechanisms across virus families.

Virus (family)	Mechanism category	Specific example(s) of resistance mechanism	Evidence/explanatory note	Key references
HIV1 (Retroviridae)	Target site mutation in viral enzymes	Mutations in reverse transcriptase (e.g., M184V, K65R), integrase (e.g., Q148H/R/K), and protease (e.g., V82A, I84V, G48V) reduce drug binding and enzymatic inhibition by NRTIs, NNRTIs, INSTIs, and PIs.	These mutations are selected under prolonged drug pressure and can lead to cross resistance within classes, necessitating resistance guided regimen changes.	[16, 57]
Influenza A virus (Orthomyxoviridae)	Target site mutation in viral surface enzyme	Neuraminidase mutations (e.g., H275Y) reduce binding of neuraminidase inhibitors such as oseltamivir and zanamivir.	Clinically resistant strains show reduced drug susceptibility and may compromise prophylaxis and treatment efficacy, especially in high risk groups.	[57, 61]
SAR‐CoV‐2 (Coronaviridae)	Target site mutation in RdRp and protease	Substitutions in RdRp (e.g., E802D, C799F) and 3CLprotease (nirmatrelvir target) can reduce susceptibility to RdRp inhibitors and protease inhibitors.	Mutations often arise under prolonged antiviral exposure in immunocompromised hosts and may also synergize with immune evasion spike variants.	[62, 65]
Hepatitis C virus (Flaviviridae)	Targetsite mutation in nonstructural proteins	Mutations in NS3/4A (e.g., A156T/V, D168A/V), NS5A (e.g., L31M/V, Y93H/N), and NS5B (e.g., S282T) reduce binding of DAAs such as protease, NS5A, and polymerase inhibitors.	Even with high efficacy DAA regimens, these resistance associated substitutions can compromise re‐treatment success and highlight the need for combination therapy and resistance testing in selected patients.	[64]

Table 6 synthesizes key drivers and risk factors for antiviral resistance from the included studies, revealing that suboptimal dosing, poor adherence, and treatment interruptions are recurrent themes across HIV, HCV, influenza, and SARS‐CoV‐2. Furthermore, the results highlight that prolonged therapy in immunocompromised hosts, over‐the‐counter or empirical use of antivirals, and low‐genetic‐barrier drugs greatly promote the selection of resistant variants. These findings underscore how human behavioral and clinical practice factors, rather than viral evolution alone shape resistance landscapes, reflecting patterns seen in bacterial AMR and emphasizing the need for integrated stewardship, diagnostics‐guided treatment, and resistance‐informed regimens within broader AMR frameworks.

**Table 6:** Drivers and risk factors of antiviral resistance.

Driver/risk factor category	Example virus(es) affected	Specific risk factor(s)	Explanatory note	Key references
Suboptimal dosing or incomplete regimens	HIV, influenza, SARS‐CoV‐2, HCV	Low dose or short course therapy, split dose regimens, prophylaxis with subtherapeutic levels	Incomplete viral suppression increases the window for resistant variants to emerge and become dominant.	[16, 66, 67]
Poor adherence and treatment interruptions	HIV, HCV	Missed doses, self‐stopping therapy, long‐term interruptions in ART or DAA regimens	Irregular drug exposure creates intermittent selection pressure and favors resistant mutants over time.	[68, 69]
Prolonged therapy and high risk hosts	HIV, SARS‐CoV‐2, influenza	Immunocompromised patients, hematopoietic stem cell or solid organ transplant recipients, patients on long‐term antiviral, suppressive therapy	Extended viral replication under drug pressure, combined with impaired immune clearance, raises the probability of resistance emergence.	[66, 67]
Over the counter or self‐medication/misuse	Influenza, SARS‐CoV‐2	Unsupervised or off‐label use of NAIs, RdRp, or protease inhibitors, empirical prophylaxis	Empirical or “just in case” use without virological confirmation or dosing oversight fuels low level drug pressure and resistance selection.	[66, 67, 69]
Virus intrinsic evolutionary traits	HIV, influenza, SARS‐CoV‐2, HCV	High replication rate, error prone RNA dependent polymerase, genetic reassortment/recombination	Inherent genetic plasticity of RNA viruses facilitates rapid generation of genetic diversity and selection of resistance‐associated mutations under drug pressure.	[32, 60]
Baseline resistance and preexisting variants	HIV, HCV	Pretreatment resistance associated substitutions or transmitted drug resistant strains	Already circulating resistant lineages can be transmitted and lead to early treatment failure even in naïve patients.	[16, 70]
Low genetic barrier drugs	HIV (lamivudine, earlier NRTIs), influenza (adamantanes, some NAIs), HCV (firstgen DAAs)	Monotherapy or single drug dominance, low‐barrier regimens allowing rapid single‐step resistance	When only one or few mutations suffice to confer resistance, resistant variants arise quickly during treatment.	[32, 69, 71–73]

## 4. Discussion

The aim of the study was to evaluate the research landscape of antiviral resistance to reveal trends, gaps, evolution, challenges and suggest future directions.

### 4.1. Growth of Literature on Antiviral Resistance

Within the 36 years (1991 to March 23, 2026) under review, 314 documents were identified on antiviral resistance with a compounded annual growth of 2%. Compared with previous bibliometric studies that examined AMR in a broad manner, our number of publications was comparatively lower. Fang et al. [71] examined AMR from a One Health perspective but did not discriminate between viral or bacterial resistance and reported 11,964 documents between 1969 and 2023. On the other hand, Sun et al. [72] examined antibacterial resistance from 2010 to 2020, and reported 2823 documents from 116 countries. In another study, Uteh et al. [73] examined AMR and mental health and reported 3449 documents between 2004 and 2025. The observed difference in the number of documents between this study and previously reported studies is likely due to differences in scope as those studies examined the broader subject of AMR. It is also important to further state that our findings are not directly comparable with previous studies. In particular, Uteh et al. [73] differed in terms of scope, database used, search strategy, inclusion criteria, and study periods. Earlier studies either examined AMR more broadly or focused primarily on antibacterial resistance, whereas the present study specifically investigated AVR [71–73]. As a result, the lower number of studies identified indicates two scenarios.

First, it indicates that though AVR contributes to the AMR discourse, it receives less attention. Thus, there is a need for more studies on AVR. Second, the relatively small number of documents may partly reflect the narrower search strategy and study scope adopted in the present study. Specifically, One Health–related terms were intentionally incorporated as mandatory components of the search strategy to identify studies addressing the interconnected human, animal, and environmental dimensions of AVR. Although this approach may have enhanced the specificity of the retrieved literature in accordance with the study′s objectives, it also represents an important methodological limitation because relevant antiviral resistance publications that did not explicitly use One Health terminology or framing may have been excluded. Thus, the final dataset of identified 314 documents should be interpreted as representing the One Health–oriented antiviral resistance literature captured by the predefined search strategy rather than the entire global antiviral resistance literature. This limitation may have influenced the observed publication volume, trends, and thematic patterns and should be considered when comparing the findings with broader AMR or antiviral resistance bibliometric studies.

Regardless of the scenarios assumed, the findings suggest that AVR remains comparatively underrepresented within the broader AMR literature, despite its growing public health significance. This is further corroborated by the growth rate observed in AVR research, which indicates a slower start for AVR research compared with ABR study by Sun et al. [72] who reported an early phase with over 80 cumulative documents in the first year. This can be attributed to a higher interest in ABR research compared with AVR. Even though the early years indicate minimal research activities, a marked shift was seen from 2002, introducing the start of a steady growth phase that showed an almost exponential trajectory. As revealed by the incorporated quadratic equation and regression analyses, there was an overall weak positive correlation between number of studies and year of publication. The number of citations failed to show a steady increase in number over the years, correlating with the minimal research outputs across the years.

### 4.2. Most Cited Studies on Antiviral Resistance

Thigpen et al. [39] focused on antiretroviral effectiveness among heterosexual individuals/couples and reported its effectiveness in prevention of transmission of HIV in this sexually active cohort. Herfst et al. [40] focused on airborne transmission of influenza A/H5N1 virus between ferrets and reported no fatality among the ferrets that were infected, and sensitivity of the virus to the antiviral drug oseltamivir. In their study, Itoh et al. [41] suggested that CA04, a H1N1 variant was sensitive to approved and experimental antiviral drugs, including zanamivir and oseltamivir. Peiris et al. [42] reviewed the ecology and evolution of highly pathogenic avian influenza H5N1 viruses and submitted that a pandemic from H5N1 is a low‐probability event but one with a potential high impact for human and public health in general. Powers and Logue [43] examined the changing patterns of Chikungunya virus re‐emergence. Alakunle et al. [44] focused on Mpox where they examined transmission, evolution, and host interactions, highlighting genetic changes, therapeutic options, and unresolved questions such as identifying its definitive animal reservoir. In their study, Sigal et al. [45] examined how HIV persists despite antiretroviral therapy through multiple infections per cell and cell‐to‐cell transmission using models, and showed that the adopted mechanism reduces drug sensitivity without requiring resistance mutations, allowing intermittent replication even at clinical drug levels.

Sturm‐Ramirez examined the role of ducks in the maintenance of H5N1 endemicity, whereas De Weerd and Nguyen [47] examined the distribution and regulation of interferons. Webster and Govorkova [48] focused on the evolving challenges of influenza virus, including the need for a universal vaccine, whereas Wong and Yuen [54] focused on avian influenza in humans. Zhou et al. [49] evaluated the biological characteristics of H7N9. Elsewhere, Dorenbaum et al. [50] examined the effectiveness of nevirapine and standard antiretroviral therapy via randomized control trial to evaluate their effectiveness to reduce perinatal HIV transmission, whereas Dybul et al. [51] published the guidelines for using antiretroviral agents among HIV‐infected persons. Bosco‐Lauth et al. [52] conducted experimental infections of dogs and cats with SARS‐CoV‐2 to understand various aspects of its biology. Agostini et al. [53] examined the antiviral properties of *β*‐d‐*N* ^4^‐hydroxycytidine against coronavirus, and found out the compound inhibits a proofreading activity. Wu et al. [55] evaluated the vertical transmission HIV escape variants vertically. Will [56] worked on a variant of prion disease. Irwin et al. [57] described antiviral drug resistance as an adaptive process. Overall, these studies were skewed in favor of various aspects of the different viruses laying a solid foundation for our understanding of AVR.

### 4.3. Keywords Analyses

Analysis of keywords can reveal the trends, evolution, and gaps in research in a particular area of study. Overall, the keyword analysis used in AVR research reflects a field that is not only strongly anchored in the concept of resistance, but one that is uneven and not comprehensively explored. The prominence of pathogens, including HIV, influenza, and COVID‐19 and specific medications, including oseltamivir, baloxavir and antiviral drugs indicates that AVR is largely concentrated on high‐burden viral infections with sustained global attention. Within this research landscape, resistance‐specific terms, including antiviral resistance, drug resistance, HIV drug resistance, and drug resistance mutations, highlight a clear focus on the emergence and mechanisms of reduced drug efficacy as exemplified by HIV‐related research on resistance [74–77]. However, the coexistence of broad and highly specific descriptors points to inconsistencies in keyword usage or studies, which may fragment the intellectual structure of the field. Moreover, the prominence of drug‐specific terms suggests that antiviral resistance research may be influenced by therapeutic applications, reflecting research priorities associated with clinically important antiviral agents. This is further reflected in temporal trends, where sustained attention to HIV‐1 is more evident, and contrasts with shorter, outbreak‐driven spikes linked to SARS‐CoV‐2, indicating that resistance research is frequently driven by immediate public health pressures instead of long‐term, anticipatory frameworks.

Thematic evolution further underscores how resistance has shifted from a generalized concept to a more specialized and pathogen‐centered focus as indicated by the concentration of SARS‐CoV‐2, various strains of influenza virus, HIV, and hepatitis virus as the core pathogens for which resistance has received greater attention. This leaves out so many pathogens, especially those that infect animals other than avian influenza virus. The absence of pathogens such as Lassa and mpox virus is notable, as recent studies have indicated resistance in these pathogens [78, 79]. Earlier research phases were characterized by broad concerns with drug resistance and pandemics, which over time evolved into more defined themes such as HIV‐1, influenza virus, and specific forms of drug resistance. This progression reflects increasing scientific maturity, particularly in understanding resistance at the molecular level, where mutation has become a central and enduring concept. However, this molecular emphasis also suggests a narrowing of perspective, with less integration of clinical, ecological, and policy‐related drivers of resistance. The continued dominance of HIV‐related resistance within evolving themes indicates a strong path dependency, where insights from one virus shape the broader research agenda, potentially limiting applicability to other viral systems.

Additionally, although concepts such as One Health and antimicrobial stewardship are emerging themes within the antiviral resistance research landscape, their prominence in the present analysis should be interpreted cautiously because One Health–related terms were mandatory components of the search strategy. Consequently, the observed clustering of these concepts with antiviral resistance is likely a reflection of predefined search criteria utilized in this study rather than their independent emergence as dominant themes in the field. Overall, the evolution of AVR research demonstrates significant advances in identifying and characterizing resistance mechanisms, but also reveals persistent gaps in integration, standardization, and long‐term strategic focus. Addressing antiviral resistance effectively will require not only deeper molecular insights but also stronger alignment with interdisciplinary and forward‐looking frameworks that capture the complexity of resistance across different viruses and contexts.

### 4.4. Journal Destinations

Journal outlets for AVR research showed authors′ preference for a few journals. The Top 10 journal outlets accounted for 28.03% of the publications, indicating authors′ preference for these outlets. The preference for a few outlets by some authors may have been driven by the quartile rankings of the journals, which were all within Q1–Q2, and impact factors. Quartile rankings of a journal relative to others within the same subject category are usually based on citation‐based performance indicators, such as impact factor or CiteScore, and serve as indicators of the journal′s relative influence, performance and visibility [79]. On the other hand, the impact factor is a journal‐level metric that mirrors the average citation performance of articles published in a journal and is widely used to assess the journal′s scientific influence among its peers [80]. These factors have been shown to influence the choice of outlets by authors as they are perceived to enhance visibility [81]. Further analyses of these outlets also showed the presence of virology, multidisciplinary and core journals. The core journals included outlets whose main scope includes resistance. The spread of journals aligns with previous reports that also showed the presence of diverse journal outlets [22, 27].

### 4.5. Institutional Analyses

Analyses of top institutions conducting AVR research from across the globe revealed a few gaps and potential challenges. First, universities from resource‐poor settings, particularly in Africa were not represented among the Top 10 institutions. Second, among the Top 10 institutions, none was a dedicated research institution focusing solely on viral resistance. The absence of institutions or universities focusing on AVR research may not necessarily be due to the absence of research facilities and funding access but may reflect the fact that AVR under the One Health framework has not occupied a unique research niche like AMR. This dominance of income‐rich countries aligns with previous studies that showed the abundance of income‐rich countries from the West [22, 27]. The dominance of the United States also aligns with the number of SCP and MCP correspondence authors and their citations as well. Setting up a research institute dedicated to AVR would not only shore up AVR research globally but will also enhance our understanding of AVR just like it is done for zoonotic diseases. For instance, the Irua Specialist Hospital in Nigeria focuses solely on this Lassa virus research [26]. The burden of HIV and its drug resistance is more in developing countries in Africa. Countries in sub‐Saharan Africa have a disproportionately higher burden than those from other regions [74–77]. Thus, setting up of specialist center for AVR is urgently needed.

### 4.6. Narrative Synthesis of AVR Resistance Mechanisms Across Diverse Viral Families, Drivers, and Risks Factors

Available evidence indicates that AVR observed across major RNA viruses is majorly driven by target‐site mutations in key viral enzymes, with consistent patterns observed among pathogens, including HIV‐1, influenza A virus, SARS‐CoV‐2, and HCV. Although these major RNA viruses differ in terms of structure and life cycles, the observed resistance converges mechanistically through nucleotide substitutions that reduce drug binding to critical proteins, including reverse transcriptase, integrase, and protease in HIV; neuraminidase in influenza; RdRp and protease in SARS‐CoV‐2; and NS3/4A, NS5A, and NS5B in HCV [16, 61, 62, 64]. This convergence in mechanisms highlights a fundamental limitation of antiviral therapeutic strategies in relying heavily on inhibitions of specific enzymes, such that even single amino acid substitutions, including M184V in HIV or H275Y in influenza can significantly negatively hinder therapeutic efficacy [59, 61]. Clinically, the impact varies across the major viral groups. For instance, HIV resistance often leads to sustained virological failure requiring regimen modification, influenza resistance may reduce treatment effectiveness and facilitate transmission, and HCV resistance is primarily relevant in re‐treatment settings due to the high efficacy of modern DAA combinations [60, 64].

Although the present synthesis predominantly focused on HIV, influenza virus, SARS‐CoV‐2, and HCV because these accounted for the majority of the retrieved publications, AVR is not restricted to these viral families. Resistance has been well documented in other clinically important viruses, including hepatitis B virus [82], herpes simplex virus [83], cytomegalovirus [84], varicella‐zoster virus [85], respiratory syncytial virus [86] and, more recently, mpox virus [78]. The limited representation of other viral families likely reflects the current publication landscape and the study′s search strategy rather than their clinical importance. Thus, future bibliometric and systematic reviews focusing on these viral families would provide a more balanced and comprehensive understanding of the evolving landscape of antiviral resistance across diverse viral pathogens.

Furthermore, evidence shows that the emergence of resistance is strongly driven by treatment practices and host‐related factors, rather than viral evolution alone. Studies indicate that suboptimal dosing, poor adherence to medication regimens, and treatment interruptions consistently create conditions for the selection of resistant variants by allowing viral replication under partial drug pressure [16, 67, 70]. These drivers are common in chronic infections, including HIV and HCV, where prolonged therapy increases not only the likelihood of resistance but also cumulative selection pressure, and in SARS‐CoV‐2, in which RAMs have been observed in immunocompromised individuals undergoing extended antiviral exposure [62, 65]. In addition, the use of antiviral drugs with a low genetic barrier to resistance also facilitates rapid emergence of escape variants, reinforcing the importance of combination therapies as stressed by recent studies [32, 69]. Intrinsic viral characteristics, including high replication rates and error‐prone polymerases, further accelerate resistance development across diverse viruses [32, 60]. The contribution of baseline or transmitted resistance, particularly in HIV, also complicates treatment outcomes and highlights the importance of resistance testing prior to therapy initiation [70].

Overall, the bibliometric findings in this study, complemented by the narrative synthesis, demonstrate that AVR is a multifactorial and system‐level challenge. The prominence of themes related to antiviral use, resistance mechanisms, surveillance, and stewardship across the retrieved literature underscores the need for integrated strategies that combine optimized drug design, improved treatment adherence, enhanced surveillance, and antimicrobial stewardship to limit the emergence, spread and impact of antiviral resistance.

## 5. Conclusion

The study assessed the research landscape of antiviral resistance globally using a combination of bibliometric and narrative synthesis. Despite the clinical and public health significance of antiviral resistance, the global importance of the AVR remains relatively underexplored compared with broader AMR research, particularly bacterial resistance. Overall, the field has witnessed modest growth, indicating limited attention compared with bacterial resistance and the need for more AVR research. At the moment, existing studies on AVR are concentrated on a few high‐burden viruses such as HIV, influenza, HCV, and SARS‐CoV‐2, leaving many other clinically and regionally relevant viruses underexplored. In addition, there exist pronounced geographical and institutional disparities in the AVR research landscape, as research output is dominated by resource‐high nations, whereas low‐ and middle‐income nations that bear one of the highest burdens of AMR, including AVR remain underrepresented due to constraints, including inadequate funding and infrastructure. Although there has been significant progress in our understanding of the molecular mechanisms of AVR, particularly mutation‐driven reductions in drug efficacy, the field remains narrowly focused, with limited integration of clinical, ecological, and One Health–driven data. Synthesized data indicate that AVR emergence is multifactorial and driven by factors, including viral evolution, poor adherence, suboptimal dosing, and prolonged drug exposure, highlighting its multifactorial nature. To address AVR effectively, a few things need to be done. First, a more inclusive, interdisciplinary, and forward‐looking approach that will holistically examine more than the reported viral pathogens is urgently needed. Genomic surveillance to monitor RNA/DNA mutations with a focus on emerging AVR genes will provide early detection/warning. There is a need to strengthen global research capacity to reduce science, technology and innovation inequality among nations for a holistic approach to ARV in the context of AMR.

## 6. Limitations

The present study has some limitations that should be acknowledged. First, we utilized only the Scopus dataset given the 100% overlap with the PubMed dataset. In addition, we included only articles and reviews written in English. Furthermore, the mandatory inclusion of One Health–related terms in the search strategy may have excluded relevant AVR publications that did not explicitly adopt a One Health framework, potentially limiting the comprehensiveness of the retrieved literature. Although this enhanced the identification of interdisciplinary studies relevant to AVR, it may also have introduced search‐strategy bias by favoring studies explicitly adopting a One Health perspective, and at the same time, led to the underrepresentation of relevant studies that did not use such terminology. As a result, the total number of retrieved documents and the prominence of One Health–related themes should be inferred within the context of the study′s predefined search strategy. By leaving out other databases, limiting the search using One Health framework and studies in other languages, some studies may have been excluded in the process. However, our findings have identified gaps that will need to be explored further.

## Author Contributions

Conceptualization: Uwem Okon Edet, Md Zulfekar Ali, and Clement Meseko; investigation, methodology, software, and validation: Md Zulfekar Ali and Uwem Okon Edet; formal analysis: Md Zulfekar Ali; data curation: Uwem Okon Edet, Md Zulfekar Ali, Bridget Enuwa Abah, Ebere Roseann Agusi, Olanrewaju Igah, Adeboyejo Kazeem Adeyinka and Clement Meseko; supervision: Uwem Okon Edet and Md Zulfekar Ali; original draft: Uwem Okon Edet and Md Zulfekar Ali; review and editing: Bridget Enuwa Abah, Ebere Roseann Agusi, Olanrewaju Igah, Adeboyejo Kazeem Adeyinka, and Clement Meseko. Uwem Okon Edet and Md Zulfekar Ali: equal contribution.

## Funding

No funding was received for this manuscript.

## Disclosure

All authors have approved the study.

## Ethics Statement

Ethical approval is not necessary for this study as it is a bibliometric study based on published literature.

## Conflicts of Interest

The authors declare no conflicts of interest.

## Data Availability

The data that support the findings of this study are available from the corresponding authors upon reasonable request
